# Memory-Enhancing Effect of a Phytosome Containing the Combined Extract of Mulberry Fruit and Ginger in an Animal Model of Ischemic Stroke with Metabolic Syndrome

**DOI:** 10.1155/2020/3096826

**Published:** 2020-07-28

**Authors:** Jintanaporn Wattanathorn, Nut Palachai, Wipawee Thukham-mee, Supaporn Muchimapura

**Affiliations:** ^1^Department of Physiology, Faculty of Medicine, Khon Kaen University, Khon Kaen, Thailand 40002; ^2^Integrative Complementary Alternative Medicine Research, Khon Kaen University, Khon Kaen, Thailand 40002; ^3^Research Institute for Human High Performance and Health Promotion, Khon Kaen University, Khon Kaen, Thailand 40002; ^4^Department of Physiology and Graduate School (Neuroscience Program), Faculty of Medicine, Khon Kaen University, Khon Kaen, Thailand 40002

## Abstract

The prevalence of dementia following cerebral ischemia in metabolic syndrome (MetS) condition is increasing, and most of the cases are often severe. Unfortunately, no effective strategy for treating this condition is available. Based on the positive modulation effect of a polyphenol-rich substance on dementia and the improvement in bioavailability and stability of polyphenols induced by the phytosome technique together with the use of the synergistic concept, we hypothesized that a phytosome containing the combined extract of mulberry fruit and ginger (PMG) should mitigate dementia and memory impairment following ischemic stroke in MetS. MetS was induced in male Wistar rats weighing 180-200 g by exposure to a 16-week feeding period of high-carbohydrate high-fat (HCHF) diet. MetS rats were orally given PMG at doses of 50, 100, and 200 mg·kg^−1^ BW 21 days before and 21 days after the occlusion of the right middle cerebral artery (Rt. MCAO). Then, their spatial memory was determined and the possible underlying mechanisms explored via the alterations of acetylcholinesterase (AChE), neuron density, malondialdehyde (MDA), superoxide dismutase (SOD), catalase (CAT), glutathione peroxidase (GSH-Px), interleukin-6 (IL-6), and signal transduction via extracellular signal-regulated kinase (ERK) pathway in both the cerebral cortex and the hippocampus. It was found that PMG significantly enhanced memory. It also decreased AChE, IL-6, and MDA but increased SOD, CAT, GSH-Px, neuron density, and phosphorylation of ERK. These data suggested the cognitive enhancing effect of PMG. The possible underlying mechanisms might occur partly via the improvement of cholinergic function via the ERK pathway together with the decrease in neurodegeneration induced by the reduction of oxidative stress and inflammation. However, a subchronic toxicity study is also required to assure the safety of PMG consumption before moving forward to a clinical trial study.

## 1. Introduction

Metabolic syndrome (MetS), a complex disorder consisting of hyperglycemia, hypertension, hypertriglyceridemia, low high-density lipoprotein cholesterol (HDL-C) levels, and central obesity [1], increases the risks of both memory impairment [2] and brain dysfunction such as stroke [3]. It has been shown that MetS raises the risk of dementia [4–6]. The underlying mechanism of memory impairment in MetS appears to be associated with the reduction of many factors including the function of the cholinergic system and signal transduction via the ERK pathway together with the reduction of neuron density in the hippocampus [6]. Recently, it has been demonstrated that stroke in MetS can also induce memory impairment [7]. The underlying pathophysiology of the aforementioned condition is also associated with a reduction of the cholinergic system and neuron density in the hippocampus but with an increase in oxidative stress and inflammation [7]. Since memory impairments in both MetS and MetS with stroke are associated with inflammation and the reduction of neuron density, the cognitive enhancing effect of substances possessing anti-inflammatory and neuroprotective effects have gained attention.

Accumulative lines of evidence have demonstrated that many plant extracts containing polyphenolic compounds including ripened mulberry (*Morus alba* Linn) fruit and the rhizome of ginger (*Zingiber officinale* Roscoe) exhibit antioxidant, anti-inflammatory, and neuroprotective properties [6, 8–11]. Unfortunately, most phenolic compounds are unstable and poorly absorbed resulting in poor bioavailability [12, 13]. However, the poor stability, short shelf life, and poor bioavailability of the polyphenols can be improved by the phytosome technique [14–18]. Therefore, we have developed a phytosome containing the combined extract of mulberry fruit and ginger (PMG). It shows higher anti-inflammatory and antioxidant activities than the conventional formulation of the extract [19]. Due to the crucial roles of inflammation and oxidative stress on the pathophysiology of memory impairment in MetS and MetS with stroke together with the anti-inflammatory and antioxidant effects of PMG, we hypothesized that PMG might be able to improve memory impairment following stroke in MetS condition. To test this hypothesis, we aimed to determine the cognitive enhancing effect of PMG in an animal model of ischemic stroke with MetS. The alterations of inflammation, oxidative stress, AChE, and signal transduction via the ERK pathway were also investigated to explore the possible underlying mechanisms.

## 2. Materials and Methods

### 2.1. Preparation of PMG

Ripened fruits of mulberry and ginger rhizomes were harvested during December 2018–January 2019. The authentication was performed by Associate Professor Panee Sirisa-ard, a pharmacognosy expert from the Faculty of Pharmacy, Chiang Mai University, who served as a consultant for the Research Institute for Human High Performance and Health Promotion, Khon Kaen University, Thailand. Voucher specimens (Nos. 61001 and 0002402) were deposited at the Integrative Complementary Alternative Medicine Research and Development Center, Khon Kaen University. The extracts of both plants and the phytosomes containing the extracts of both plants were prepared as mentioned in a previous study [19]. The concentrations of active ingredients including total phenolic compounds, flavonoids, gingerol, cyanidin-3-O-glucoside, quercetin-3-rutinoside, ferulic acid, and gallic acid together with the biological activities including antioxidant, anti-inflammatory, and lipase-suppressing activities are similar to those mentioned in our previous study [19].

### 2.2. Experimental Protocol

The experimental animals used in this study were male Wistar rats, weighing 180-220 g and aged 8 weeks old, from the National Laboratory Animal Center, Salaya, Nakhon Pathom, Thailand. They were housed in standard metal cages (6 per cage) in standard laboratory conditions (23 ± 2°C, 12 : 12 hour light : dark cycle). They were provided with food and water ad libitum. All procedures and experimental protocols were approved by the Institutional Animal Ethics Committee of Khon Kaen University (record no. IACUC-KKU 95/60). After a one-week acclimatization period, rats were randomly divided into 8 groups (*n* = 6) described as follows:

1. Group I (ND + vehicle): all rats in this group were fed with normal diet and treated with vehicle
2. Group II (HCHF + Sham + Vehicle): all rats in this group received a high-carbohydrate high-fat (HCHF) diet and were exposed to a surgical operation to expose the right middle cerebral artery without the occlusion of this artery and received vehicle or distilled water for treatment
3. Group III (HCHF + MCAO + Vehicle): all rats in this group were fed with a HCHF diet, subjected to the occlusion at the right middle cerebral artery (Rt. MCAO) and treated with vehicle
4. Group IV (HCHF + MCAO + Vitamin C): all rats in this group received a HCHF diet and were subjected to both Rt. MCAO occlusion and vitamin C (250 mg · kg^−1^ BW) treatment
5. Group V (HCHF + MCAO + Donepezil): rats in this group were exposed to the same condition as mentioned in (4) except that they were treated with donepezil at a dose of 3 mg · kg^−1^ BW
6. Group VI-VIII (HCHF + MCAO + PMG) (PMG50, PMG100, and PMG 200): all rats in these groups were treated as mentioned in (4) but they were treated with PMG at doses of 50, 100, to 200 mg · kg^−1^ BW

**Figure 1:**
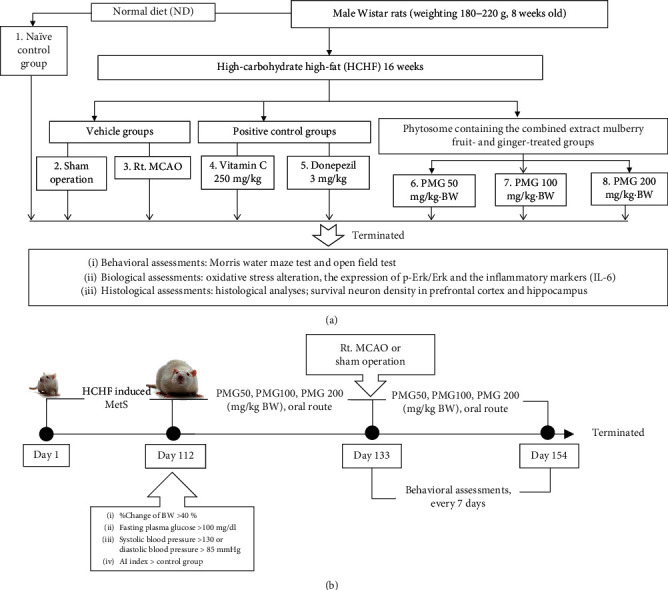
Schematic diagram showing all experimental procedures. (a) Experimental protocol of PMG treatment and the determination of various parameters. (b) Right MCAO induction and schedule for PMG treatment. IL-6: interleukin-6; P-Erk: phosphorylated-extracellular signal-regulated protein kinase; Erk: extracellular signal-regulated protein kinase; PMG50, PMG100, and PMG200: the phytosomes containing the combined extract of mulberry fruit and ginger at doses of 50, 100, and 200 mg·kg^−1^ BW, respectively.

Rats in group I were fed with a normal diet containing 4.5% fat, 42% carbohydrate, and 24% protein, whereas rats in groups II-VIII were fed with a HCHF diet containing 35% fat, 45% carbohydrate, and 20% protein to induce MetS. After 16 weeks of the feeding period, rats which showed the criteria of MetS [19] were selected for inducing ischemic stroke by Rt. MCAO. The animals were orally treated with the assigned substances once daily for 21 days before and 21 days after Rt. MCAO induction. Spatial memory and locomotor assessments were performed every 7 days throughout the study period. At the end of the study period, neuron density, oxidative stress status, AChE activity, and the expressions of ERK phosphorylation together with IL-6 in the prefrontal cortex and the hippocampus were determined. All experimental protocols were summarized in the schematic diagram shown in Figure 1.

### 2.3. Induction of Right Middle Cerebral Artery Occlusion

Focal cerebral ischemia was induced by using the ischemia/reperfusion (I/R) induction technique. After anesthetization with pentobarbital sodium at a dose of 60 mg · kg^−1^ BW, body temperature was maintained at 37.0 ± 0.5°C with the aid of a warming pad during the operation. A midline incision was made in the neck and the right common carotid artery, the external carotid artery, and the internal carotid artery were isolated. A round-tip monofilament nylon number 4.0 (1.5 metric, Reg. No. 002667, Unik Surgical Sutures Mfg. Co., New Taipei, Taiwan) coated with silicone was inserted from the right common carotid artery into the internal carotid artery and advanced forward until a faint resistance was felt in order to block the origin of the middle cerebral artery. After the exposure to a 90-minute right middle artery occlusion, the filament was withdrawn to allow reperfusion and the wound was sutured [20]. On the other hand, a sham operation was performed following the induction of a right middle cerebral artery occlusion without the step of inserting the nylon monofilament coated with silicone.

### 2.4. Assessment of Spatial Memory

Morris water maze test was assessed to measure the hippocampal-dependent memory or spatial memory [21]. In brief, the animals were trained to memorize the association between their location and the location of an immersed platform under the water in a 4-quadrant circular water pool (147 cm in diameter) which was obscured by nontoxic milk powder by using external cues. The depth of the water was 60 cm, and the temperature of the water was 25 ± 1°C. After 4 training sessions, escape latency or the time spent by each animal to find and climb onto the immersed platform was recorded. Twenty-four hours later, each animal was reexposed to the same condition except that the immersed platform was removed. The time which each animal spent swimming in the quadrant which previously contained the immersed platform or retention time was also measured.

### 2.5. Assessment of Locomotor Activity

Measurement of locomotor activity was performed by using an open field test. Each rat was placed into the center of a 90 × 70 cm square plexiglass chamber which was placed in a room lit by a 60 W light bulb 1.75 m above the center of the open field chamber. Each rat was allowed 5 minutes of exploration time. The number of times each animal entered one of the squares in the open field chamber or crossing was recorded. In addition, the number of times each rat entered into the center square and the number of exploratory activities including licking, rearing, and grooming were also recorded by using a video tracking system [22].

### 2.6. Histological Procedure and Nissl Staining

The coronal sections of the brain containing the dorsal hippocampus were prepared at 10 *μ*m thick via a cryostat (Thermo Scientific™ HM525 Cryostat). In brief, the brains were removed and fixed with 4% paraformaldehyde (Sigma-Aldrich, USA) in 0.1 M phosphate buffer pH 7.4, 4°C overnight. Then, they were infiltrated with 30% sucrose (Merck, Germany) solution for 48–72 hours. Following the mentioned process, the serial sections of the brains were prepared at 10 *μ*m thick and placed on slides coated with 0.3% aqueous solution of gelatin containing 0.05% aluminum potassium sulfate (Sigma-Aldrich, USA). Then, they were stained with 0.25% cresyl violet (Sigma-Aldrich, USA), dehydrated through graded alcohols (70, 95, 100%; 2x) (RCI Labscan, Thailand), placed in xylene (Merck, Germany), and mounted using a DPX mounting reagent (Merck, Germany). The assessment of neuron density in the prefrontal cortex and CA1, CA2, CA3 and the dentate gyrus of the hippocampus was performed under an Olympus light microscope model BH-2 (Japan) at 40x magnification. Counts were performed in three adjacent fields, and the mean number was calculated. The results were expressed as the density of neurons per 255 *μ*m^2^ [23].

### 2.7. Biochemical Assessments

The prefrontal cortex and hippocampus were isolated and prepared as a tissue homogenate with 50 volumes of 0.1 M phosphate buffer saline. Then, the brain homogenates were subjected to a 16,000 rpm centrifugation process for 5 minutes. The supernatant was harvested and used for the determination of oxidative stress status markers including MDA level and the activities of SOD, CAT, and GSH-Px. In addition, AChE activity was also determined. The protein concentration in the brain homogenate was assessed using a Thermo Scientific NanoDrop 2000c spectrophotometer (Thermo Fisher Scientific, Wilmington, DE, USA), and the optical density at a wavelength of 280 nm was measured.

#### 2.7.1. Determination of Oxidative Stress Status

Malondialdehyde was used as an indicator of lipid peroxidation and determined using a thiobarbituric acid reaction. In brief, an aliquot of tissue sample at a volume of 50 *μ*l was mixed with a mixed solution containing 8.1% sodium dodecyl sulfate (Sigma-Aldrich, USA) (50 *μ*l), 0.8% thiobarbituric acid (Sigma-Aldrich, USA) (375 *μ*l), 20% acetic acid (Sigma-Aldrich, USA) (375 *μ*l), and distilled water (150 *μ*l). After mixing thoroughly, the mixture was subjected to 95°C heat for 60 minutes. Following this process, the mixed solution was cooled with tap water and mixed with the mixture containing n-butanol and pyridine (Merck, Germany) at a ratio of 15 : 1 (1250 *μ*l) and DW (250 *μ*l). After mixing, the solution was subjected to a 4,000 rpm centrifugation process for 10 minutes. At the end of the centrifugation period, the upper layer was harvested and the absorbance at 532 nm was determined. Standard was prepared by using 1,1,3,3-tetramethoxy propane (0–15 *μ*M) (Sigma-Aldrich, USA). The level of MDA was expressed as ng/mg protein [24].

SOD activity was assessed based on the inhibition of nitroblue tetrazolium reduction. Briefly, 20 *μ*l of the harvested tissue homogenate was mixed with the reaction solution containing 57 mM phosphate buffer solution (KH_2_PO_4_) (Sigma-Aldrich, USA), 0.1 mM EDTA (Sigma-Aldrich, USA), 10 mM cytochrome *c* (Sigma-Aldrich, USA), and 50 *μ*M of xanthine (Sigma-Aldrich, USA) at a volume of 200 *μ*l. Following this process, 20 *μ*l of xanthine oxidase solution (0.90 mU/ml) (Sigma-Aldrich, USA) was added and the absorbance was measured at 415 nm. SOD enzyme (Sigma-Aldrich, USA) activities at concentrations ranging from 0 to 25 units/ml were served as standard, and the SOD activities were expressed as units/mg protein [25].

The determination of CAT was performed based on the ability of the enzyme to break down H_2_O_2_. An aliquot of the harvested tissue homogenate at a volume of 10 *μ*l was mixed with the solution comprising 30 mM hydrogen peroxide (in 50 mM phosphate buffer, pH 7.0) (BDH Chemicals Ltd., UK) (50 *μ*l), H_2_SO_4_ (Sigma-Aldrich, USA) (25 *μ*l), and 5 mM KMnO_4_ (Sigma-Aldrich, USA) (150 *μ*l). Absorbance at 490 nm was measured. The CAT enzymes (Sigma-Aldrich, USA) at concentrations ranging from 0 to 100 units/ml were served as standard, and the CAT activities were also expressed as units/mg protein [26].

Assay of glutathione peroxidase activity was carried out by mixing 20 *μ*l of the harvested tissue homogenate with a mixed solution consisting of 1 mM dithiothreitol (DTT) (Sigma-Aldrich, USA) (10 *μ*l) in 6.67 mM potassium phosphate buffer (Sigma-Aldrich, USA) (pH 7), 1 mM sodium azide (Sigma-Aldrich, USA) (100 *μ*l) in 6.67 mM potassium phosphate buffer (Sigma-Aldrich, USA) (pH 7), 50 mM glutathione solution (Sigma-Aldrich, USA) (10 *μ*l), and 30% hydrogen peroxide (BDH Chemicals Ltd., UK) (100 *μ*l). After mixing thoroughly, the solution was subjected to a 5-minute incubation period at 25°C. At the end of the incubation period, 10 mM DTNB (5,5′-dithiobis(2-nitrobenzoic acid)) (Sigma-Aldrich, USA) (10 *μ*l) was added and an absorbance at 412 nm was measured at 25°C for 5 minutes. GSH-Px enzymes (Sigma-Aldrich, USA) at concentrations ranging from 0 to 5 units/ml were used for preparing a standard calibration curve. GSH-Px activity was expressed as units/mg protein [27].

#### 2.7.2. Assessment of Acetylcholinesterase Activity

Acetylcholinesterase activity was determined by using the spectrophotometric method. In brief, the mixed reagent consisting of 0.1 mM sodium phosphate buffer (pH 8.0) (Sigma-Aldrich, USA) (200 *μ*l) and 0.2 M DTNB (5,5′-dithiobis(2-nitrobenzoic acid)) (Sigma-Aldrich, USA) (10 *μ*l) was mixed with 20 *μ*l of the harvested tissue homogenate and incubated at 25°C for 5 minutes. Following this process, 10 *μ*l of 15 mM acetylcholine thiochloride (ACTI) (Sigma-Aldrich, USA) was added and incubated for 3 minutes. At the end of the incubation period, an absorbance at 412 nm was measured by using a microplate reader (iMark Microplate Absorbance Reader). The results were calculated according to the following equation, and the activity of AChE was expressed as nmol/min·mg protein [28]:

(1) $$\begin{array}{c} \text{AChe activity}=\left(\frac{\Delta A}{1.36\times 10^{4}}\right)\times \left(\frac{1}{120/230}\right)C, \end{array}$$

where Δ*A* is the difference of absorbance/minute and *C* is the protein concentration of brain homogenate.

### 2.8. Western Blotting Analysis

Tissue homogenates of the prefrontal cortex and the hippocampus were suspended and homogenized in the Mammalian Protein Extraction Reagent (M-PER; Pierce Protein Biology Product, Rockford, IL, USA), with a protease inhibitor cocktail (1 : 10) (Sigma-Aldrich, USA). The tissue homogenate was centrifuged at 12,000 g for 10 minutes at 4°C. The supernatant was harvested and used for assessing the expressions of ERK, phosphorylation ERK, and IL-6. Protein concentration was detected using a Thermo Scientific NanoDrop 2000c spectrophotometer (Thermo Fisher Scientific, Wilmington, DE, USA). An aliquot of the sample at 60 micrograms was adjusted to an appropriate concentration using a Tris-glycine SDS-PAGE loading buffer (Bio-Rad, USA) and heated at 95°C for 10 minutes. Protein in sample was isolated via sodium dodecyl sulfate polyacrylamide gel electrophoresis (SDS-PAGE) by loading the sample at a volume of 20 *μ*l on SDS-polyacrylamide gel. Then, the separated bands were transferred to a nitrocellulose membrane, washed with 0.05% TBS-T, and subjected to a 1-hour incubation period in blocking buffer (5% skim milk in 0.1% TBS-T) at 25°C. After this process, the nitrocellulose membrane was incubated with anti-phosphoERK1/2 (Thr202/Tyr204) (Cell Signaling Technology, USA; dilution 1 : 1000), anti-ERK1/2 (Cell Signaling Technology, USA; dilution 1 : 1000), anti-IL-6 (Cell Signaling Technology, USA; dilution 1 : 500), and anti-*β*-actin (Cell Signaling Technology, USA; dilution 1 : 1000) antibodies at 25°C for 2 hours. Following an incubation process, the nitrocellulose membrane was rinsed with TBS-T (0.05%) again and subjected to a 1-hour incubation with an anti-rabbit IgG HRP-linked antibody (Cell Signaling Technology, USA; dilution 1 : 2000) at 25°C. The ECL detection systems (GE Healthcare) and LAS 4000 luminescent image analyzer (GE Healthcare) were used for visualizing and quantifying the bands. Band intensities were measured for statistical analysis using the ImageQuant TL v.7.0 image analysis software (GE Healthcare). The expression was normalized using anti-total-ERK1/2 and anti-*β*-actin for ERK1/2 and IL-6, respectively. Data were presented as a relative density to the naïve control group [19].

### 2.9. Statistical Analysis

All data are presented as mean ± standard error of mean (SEM). Statistical significance was evaluated by using one-way analysis of variance (ANOVA), followed by the post hoc (Tukey) test. Statistical significance was regarded at *p* value < 0.05. All statistical data analyses were performed using SPSS version 21.0 (released 2012; IBM SPSS Statistics for Windows, IBM Corp.).

## 3. Results

### 3.1. Cognitive Enhancing Effect of PMG

**Figure 2:**
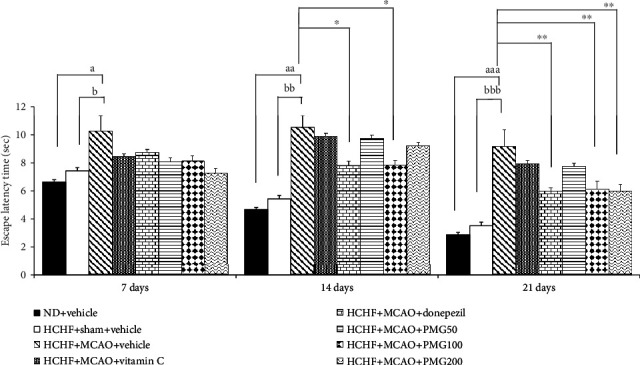
Effect of PMG on escape latency time under cognitive deficit induced by Rt. MCAO. Data are presented as mean ± SEM (*n* = 6/group). ^a^*p* value < 0.05, ^aa^*p* value < 0.01, and ^aaa^*p* value < 0.001; compared between naïve control which received a normal diet and vehicle and MetS+MCAO rats which received HCHF, MCAO, and vehicle. ^b^*p* value < 0.05, ^bb^*p* value < 0.01, and ^bbb^*p* value < 0.001; compared between sham rats which received HCHF, sham operation, and vehicle and MetS+MCAO rats which received HCHF, MCAO, and vehicle. ^∗^*p* value < 0.05 and ^∗∗^*p* value < 0.01; compared to MetS+MCAO rats which received HCHF, MCAO, and vehicle. ND: normal diet; HCHF: high-carbohydrate high-fat diet; MetS: metabolic syndrome; MCAO: middle cerebral artery occlusion; Vitamin C: vitamin C at a dose of 250 mg·kg^−1^ BW; Donepezil: donepezil at a dose of 3 mg·kg^−1^ BW; PMG50, PMG100, and PMG200: the phytosomes containing the combined extract of mulberry fruit and ginger at doses of 50, 100, and 200 mg·kg^−1^ BW, respectively.

**Figure 3:**
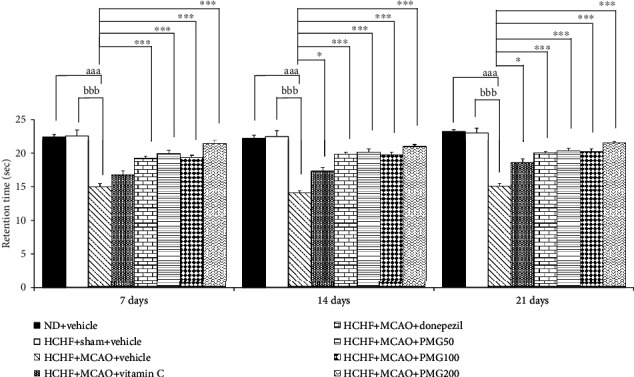
Effect of PMG on retention time under cognitive deficit induced by Rt. MCAO. Data are presented as mean ± SEM (*n* = 6/group). ^aaa^*p* value < 0.001; compared between naïve control which received a normal diet and vehicle and MetS+MCAO rats which received HCHF, MCAO, and vehicle. ^bbb^*p* value < 0.001; compared between sham rats which received HCHF, sham operation, and vehicle and MetS+MCAO rats which received HCHF, MCAO, and vehicle. ^∗^*p* value < 0.05 and ^∗∗∗^*p* value < 0.001; compared to MetS+MCAO rats which received HCHF, MCAO, and vehicle. ND: normal diet; HCHF: high-carbohydrate high-fat diet; MetS: metabolic syndrome; MCAO: middle cerebral artery occlusion; Vitamin C: vitamin C at a dose of 250 mg·kg^−1^ BW; Donepezil: donepezil at a dose of 3 mg·kg^−1^ BW; PMG50, PMG100, and PMG200: the phytosomes containing the combined extract of mulberry fruit and ginger at doses of 50, 100, and 200 mg·kg^−1^ BW, respectively.

The effect of PMG on escape latency is shown in Figure 2. Sham operation produced no significant change on escape latency in MetS which received vehicle. MetS rats induced by an HCHF diet which were subjected to MCAO and received vehicle significantly increased escape latency times at 7, 14, and 21 days after MCAO (*p* value < 0.05, 0.01, and 0.001, respectively; compared to the HCHF+sham operation+vehicle group). At 7 days after MCAO, MetS rats with MCAO which received all assigned substances failed to produce a significant modulation effect on escape latency. However, the increase in escape latency in MetS with MCAO was counteracted by donepezil and PMG at a dose of 100 mg·kg^−1^ BW (all *p* value < 0.05; compared to the HCHF+MCAO+vehicle group) at 14 days after MCAO. At 21 days after MCAO, MetS rats with MCAO which received donepezil and PMG both at doses of 100 and 200 mg·kg^−1^BW had significantly decreased escape latencies (all *p* value < 0.01; compared to the HCHF+MCAO+vehicle group).  Figure 3 shows the effect of PMG on retention time in MetS with MCAO. It was shown that sham operation failed to show the reduction in retention time but MCAO significantly induced the reduction in retention time throughout the study period (all *p* value < 0.001; compared to the HCHF+sham operation+vehicle group). Donepezil and all doses of PMG used in this study significantly counteracted the reduction of retention time in MetS rats with MCAO (all *p* value < 0.001; compared to the HCHF+MCAO+vehicle group) throughout the study period. In addition, the significant increases in retention time in MetS rats with MCAO which received vitamin C at 14 and 21 days after MCAO (all *p* value < 0.05; compared to the HCHF+MCAO+vehicle group) were also observed.

**Figure 4:**
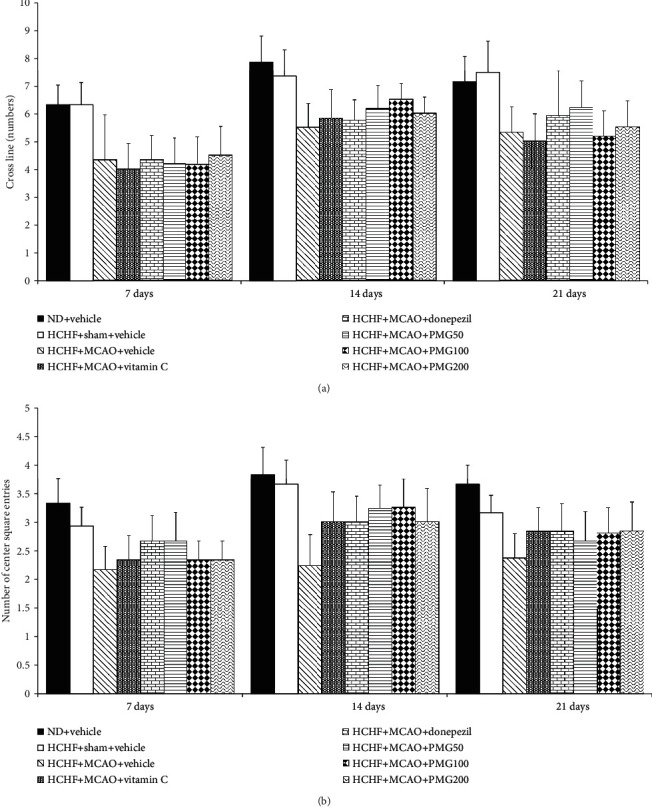
Effect of PMG on locomotor activities: (a) the number of cross lines and (b) the number of center square entries. Data are presented as mean ± SEM (*n* = 6/group). ND: normal diet; HCHF: high-carbohydrate high-fat diet; MCAO: middle cerebral artery occlusion; Vitamin C: vitamin C at dose of 250 mg·kg^−1^ BW; Donepezil: donepezil at a dose of 3 mg·kg^−1^ BW; PMG50, PMG100, and PMG200: the phytosomes containing the combined extract of mulberry fruit and ginger at doses of 50, 100, and 200 mg·kg^−1^ BW, respectively.

**Figure 5:**
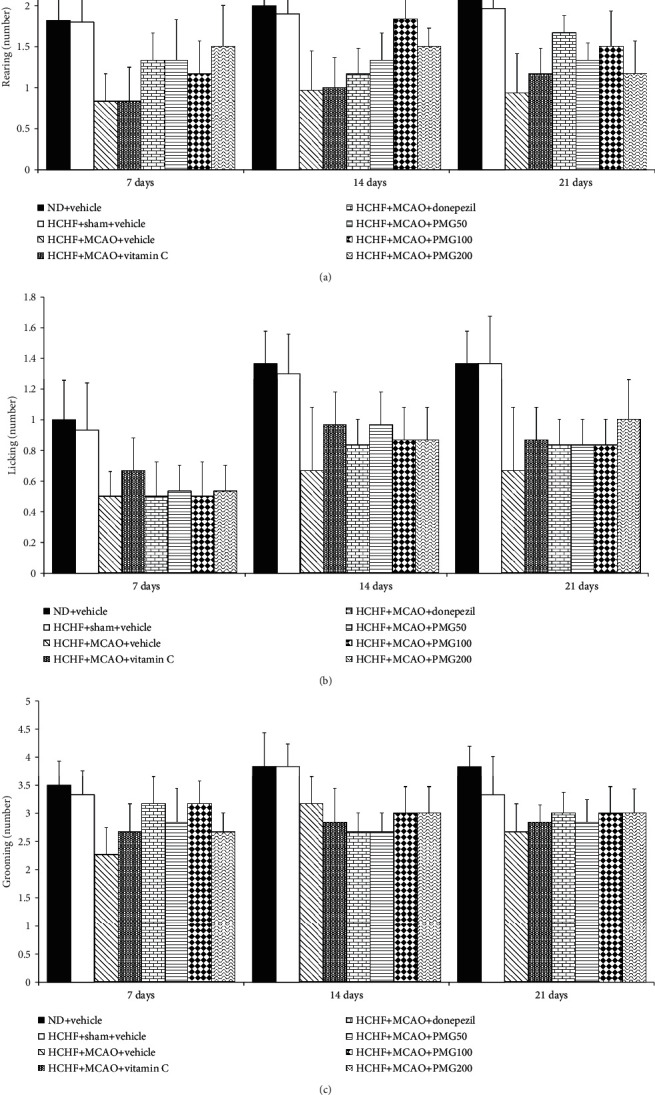
Effect of PMG on exploratory activities: (a) rearing, (b) licking, and (c) grooming behaviors. Data are presented as mean ± SEM (*n* = 6/group). ND: normal diet; HCHF: high-carbohydrate high-fat diet; MCAO: middle cerebral artery occlusion; Vitamin C: vitamin C at a dose of 250 mg·kg^−1^ BW; Donepezil: donepezil at a dose of 3 mg·kg^−1^ BW; PMG50, PMG100, and PMG200: the phytosomes containing the combined extract of mulberry fruit and ginger at doses of 50, 100, and 200 mg·kg^−1^ BW, respectively.

In order to assure that the cognitive enhancing effect mentioned earlier was not a false positive, the effects of PMG on locomotor activity and exploratory activity were also determined and the results are shown in Figures 4 and 5. The present results showed that no significant changes on locomotor activities were observed in any groups when compared to rats which receive a normal diet and vehicle.

### 3.2. Effect of PMG on the Density of Survival Neuron

**Figure 6:**
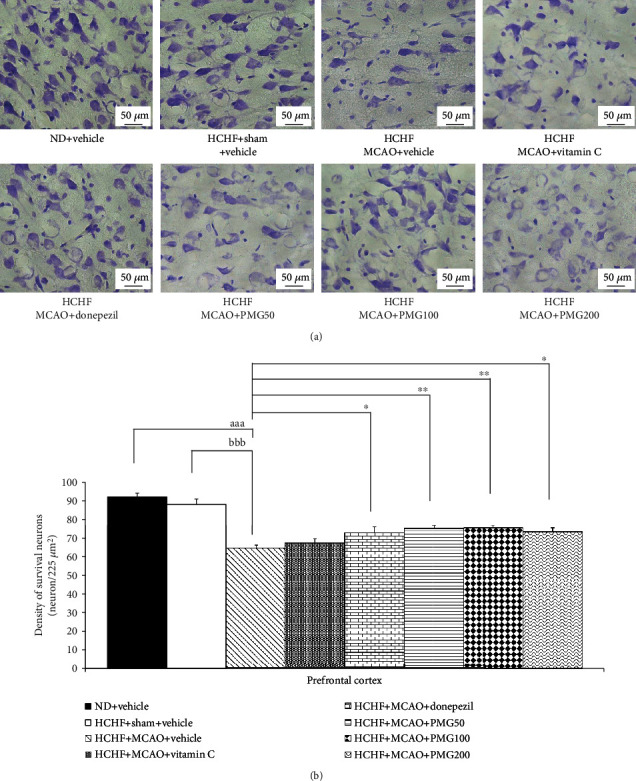
Effect of PMG on neuron density in the medial prefrontal cortex. (a) Light microscopy of coronal sections in the medial prefrontal cortex which were stained with cresyl violet at 40x magnification. (b) Density of survival neurons in the medial prefrontal cortex. Data are presented as mean ± SEM (*n* = 6/group). ^aaa^*p* value < 0.001; compared between naïve control which received a normal diet and vehicle and MetS+MCAO rats which received HCHF, MCAO, and vehicle. ^bbb^*p* value < 0.001; compared between sham rats which received HCHF, sham operation, and vehicle and MetS+MCAO rats which received HCHF, MCAO, and vehicle. ^∗^*p* value < 0.05 and ^∗∗^*p* value < 0.01; compared to MetS+MCAO rats which received HCHF, MCAO, and vehicle. ND: normal diet; HCHF: high-carbohydrate high-fat diet; MetS: metabolic syndrome; MCAO: middle cerebral artery occlusion; Vitamin C: vitamin C at a dose of 250 mg·kg^−1^ BW; Donepezil: donepezil at a dose of 3 mg·kg^−1^ BW; PMG50, PMG100, and PMG200: the phytosomes containing the combined extract of mulberry fruit and ginger at doses of 50, 100, and 200 mg·kg^−1^ BW, respectively.

**Figure 7:**
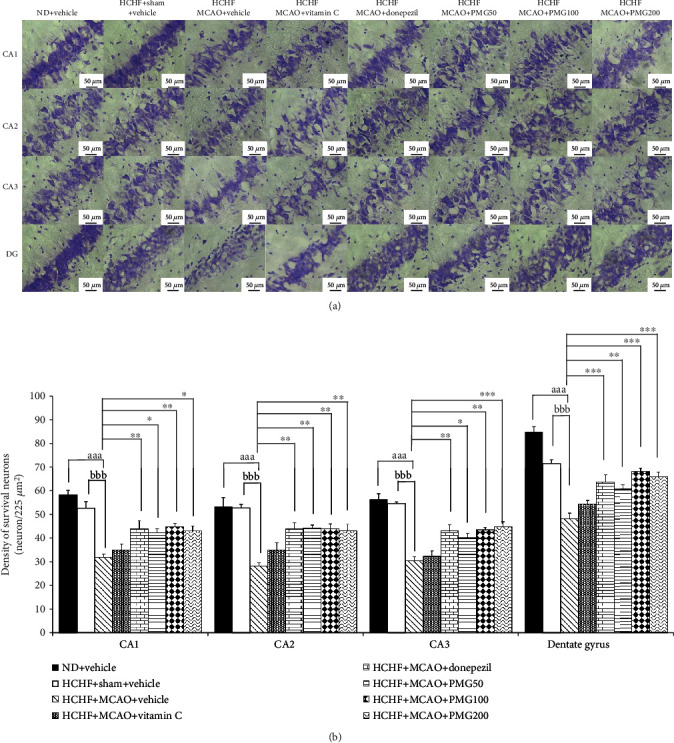
Effect of PMG on neuron density in various subregions of the hippocampus. (a) Light microscopy of coronal sections in CA1, CA2, CA3, and dentate gyrus of the hippocampus which were stained with cresyl violet at 40x magnification. (b) Density of survival neurons in CA1, CA2, CA3, and dentate gyrus of the hippocampus. Data are presented as mean ± SEM (*n* = 6/group). ^aaa^*p* value < 0.001; compared between naïve control which received a normal diet and vehicle and MetS+MCAO rats which received HCHF, MCAO, and vehicle. ^bbb^*p* value < 0.001; compared between sham rats which received HCHF, sham operation, and vehicle and MetS+MCAO rats which received HCHF, MCAO, and vehicle. ^∗^*p* value < 0.05, ^∗∗^*p* value < 0.01, and ^∗∗∗^*p* value < 0.001; compared to MetS+MCAO rats which received HCHF, MCAO, and vehicle. ND: normal diet; HCHF: high-carbohydrate high-fat diet; MetS: metabolic syndrome; MCAO: middle cerebral artery occlusion; Vitamin C: vitamin C at a dose of 250 mg·kg^−1^ BW; Donepezil: donepezil at a dose of 3 mg·kg^−1^ BW; PMG50, PMG100, and PMG200: the phytosomes containing the combined extract of mulberry fruit and ginger at doses of 50, 100, and 200 mg·kg^−1^ BW, respectively.

Based on the positive modulation effect of PMG on memory observed in this study and the crucial role of the increased neuron density either via neurogenesis or via the reduction of neurodegeneration [29], the changes of neuron density in the prefrontal cortex and the hippocampus (the areas contributing an important role on memory) were also investigated to explore the possible underlying mechanism.  Figure 6 shows the effect of PMG on neuron density in the prefrontal cortex. The sham operation failed to show a significant change of neuron density in the prefrontal cortex, whereas MCAO significantly decreased neuron density in the aforementioned area (*p* value < 0.001; compared to the HCHF+sham operation+vehicle group). However, this change was mitigated by donepezil and all doses of PMG used in this study (*p* value <0.05, 0.01, 0.01, and 0.05, respectively; compared to the HCHF+MCAO+vehicle group). The effect of PMG on neuron density in CA1, CA2, CA3, and the dentate gyrus of the hippocampus is shown in Figure 7. The sham operation also failed to produce the change in neuron density in all subregions of the hippocampus investigated in this study, while MCAO produced a significant reduction of neuron density in all subregions of the hippocampus mentioned earlier (all *p* value < 0.001; compared to the HCHF+sham operation+vehicle group). Donepezil and PMG at doses of 50, 100, and 200 mg·kg^−1^ BW significantly mitigated the reduction of neuron density in CA1 (*p* value < 0.01, 0.05, 0.01, and 0.05, respectively; compared to the HCHF+MCAO+vehicle group), CA2 (all *p* value < 0.01; compared to the HCHF+MCAO+vehicle group), CA3 (*p* value < 0.01, 0.05, 0.01, and 0.001, respectively; compared to HCHF+MCAO+vehicle group), and the dentate gyrus (*p* value < 0.001, 0.01, 0.001, and 0.001, respectively; compared to the HCHF+MCAO+vehicle group).

### 3.3. Effect of PMG on Biochemical Changes

To explore the possible underlying mechanism of PMG, the biochemical changes in both the prefrontal cortex and the hippocampus were also investigated, and results are shown in Table 1 and Table 2. The current results revealed that no significant changes in MDA, SOD, CAT, and GSH-Px activities were observed in the prefrontal cortex of MetS rats with the sham operation which received vehicle, whereas significantly decreased SOD, CAT, and GSH-Px activities and an increased MDA level were observed in MetS rats with MCAO as shown in Table 1 (*p* value < 0.01, 0.01, 0.001, and 0.001, respectively; compared to the HCHF+sham operation+vehicle group). Vitamin C significantly increased SOD and GSH-Px activities but decreased the MDA level (*p* value < 0.01, 0.05, and 0.001, respectively; compared to the HCHF+MCAO+vehicle group), whereas donepezil failed to show significant changes in all parameters reflecting oxidative stress in MetS rats with MCAO. It has been shown that a low dose of PMG significantly increased SOD and GSH-Px activities (all *p* value < 0.001; compared to the HCHF+MCAO+vehicle group) in MetS rats with MCAO. Both medium and high doses of PMG used in this study significantly increased SOD, CAT, and GSH-Px activities but decreased the MDA level (*p* value < 0.001, 0.05, 0.001, and 0.001, respectively; compared to the HCHF+MCAO+vehicle group) in MetS rats with MCAO. Table 2 shows that no significant changes of the aforementioned parameters in MetS rats with the sham operation which received vehicle were observed, but a significant reduction of SOD, CAT, and GSH-Px activities together with the elevation of the MDA level in the hippocampus of MetS rats with MCAO which received vehicle were observed (all *p* value < 0.001; compared to the HCHF+sham operation+vehicle group). Vitamin C treatment significantly increased SOD, CAT, and GSH-Px activities (*p* value < 0.01, 0.05, and 0.05, respectively; compared to the HCHF+MCAO+vehicle group) but decreased the MDA level (*p* value < 0.001; compared to the HCHF+MCAO+vehicle group) in the area just mentioned of MetS rats with MCAO. All doses of PMG significantly increased SOD and GSH-Px activities but decreased the MDA level (all *p* < 0.001; compared to the HCHF+MCAO+vehicle group) in MetS rats with MCAO. However, the elevation of CAT activity in the hippocampus of MetS rats with MCAO presented only in MetS with MCAO which received medium and high doses of PMG used in this study (all *p* value < 0.01; compared to the HCHF+MCAO+vehicle group).

The alterations of the AChE activity in the cerebral cortex and the hippocampus were also explored, and the results are shown in Tables 1 and 2. MetS rats with a sham operation which received vehicle failed to show a significant change of this parameter, but a significant elevation of AChE was observed in the prefrontal cortex and the hippocampus of MetS rats with MCAO which received vehicle (*p* value < 0.001 all; compared to the HCHF+sham operation+vehicle group). Donepezil and PMG at doses of 50, 100, and 200 mg·kg^−1^ BW mitigated the increase in AChE in both the prefrontal cortex and the hippocampus of MetS rats with MCAO (*p* value < 0.01, 0.05, 0.01, and 0.05; *p* < 0.001, 0.01, 0.001, and 0.001; compared to the HCHF+MCAO+vehicle group).

### 3.4. Change of Signal Transduction via Extracellular Recognition Kinase (ERK) Pathway in Prefrontal Cortex and Hippocampus

**Figure 8:**
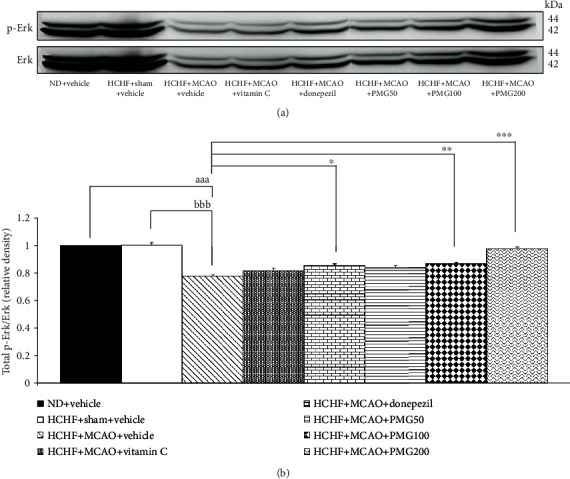
Effect of PMG on the expression of total p-Erk/Erk in the prefrontal cortex was detected by Western blotting. (a) Representative Western blot showing the levels of total p-Erk/Erk. (b) Relative density of total p-Erk/Erk. Data are presented as mean ± SEM (*n* = 6/group). ^aaa^*p* value < 0.001; compared between naïve control which received a normal diet and vehicle and MetS+MCAO rats which received HCHF, MCAO, and vehicle. ^bbb^*p* value < 0.001; compared between sham rats which received HCHF, sham operation, and vehicle and MetS+MCAO rats which received HCHF, MCAO, and vehicle. ^∗^*p* value < 0.05, ^∗∗^*p* value < 0.01, and ^∗∗∗^*p* value < 0.001; compared to MetS+MCAO rats which received HCHF, MCAO, and vehicle. ND: normal diet; HCHF: high-carbohydrate high-fat diet; MetS: metabolic syndrome; MCAO: middle cerebral artery occlusion; Vitamin C: vitamin C at a dose of 250 mg·kg^−1^ BW; Donepezil: donepezil at a dose of 3 mg·kg^−1^ BW; PMG50, PMG100, and PMG200: the phytosomes containing the combined extract of mulberry fruit and ginger at doses of 50, 100, and 200 mg·kg^−1^ BW, respectively.

**Figure 9:**
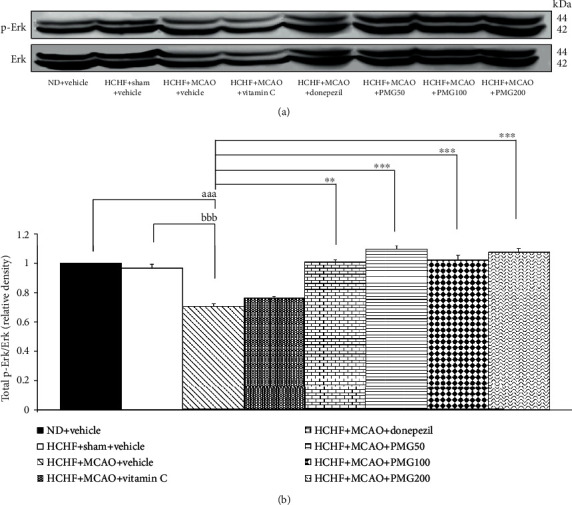
Effect of PMG on the expression of total p-Erk/Erk in the hippocampus was detected by Western blotting. (a) Representative Western blot showing the levels of total p-Erk/Erk. (b) Relative density of total p-Erk/Erk. Data are presented as mean ± SEM (*n* = 6/group). ^aaa^*p* value < 0.001; compared between naïve control which received a normal diet and vehicle and MetS+MCAO rats which received HCHF, MCAO, and vehicle. ^bbb^*p* value < 0.001; compared between sham rats which received HCHF, sham operation, and vehicle and MetS+MCAO rats which received HCHF, MCAO, and vehicle. ^∗∗^*p* value < 0.01 and ^∗∗∗^*p* value < 0.001; compared to MetS+MCAO rats which received HCHF, MCAO, and vehicle. ND: normal diet; HCHF: high-carbohydrate high-fat diet; MetS: metabolic syndrome; MCAO: middle cerebral artery occlusion; Vitamin C: vitamin C at a dose of 250 mg·kg^−1^ BW; Donepezil: donepezil at a dose of 3 mg·kg^−1^ BW; PMG50, PMG100, and PMG200: the phytosomes containing the combined extract of mulberry fruit and ginger at doses of 50, 100, and 200 mg·kg^−1^, respectively.

Based on the important role of ERK signal transduction on brain plasticity and memory enhancement [30], the effects of PMG on ERK phosphorylation in the prefrontal cortex and the hippocampus were also assessed, and results are shown in Figures 8 and 9. The current data showed that MCAO induction in MetS rats significantly decreased the expressions of ERK phosphorylation in both the prefrontal cortex and the hippocampus (all *p* value < 0.001; compared to the HCHF+sham operation+vehicle group). However, donepezil and PMG treatment at doses of 100 and 200 mg·kg^−1^ BW mitigated the reduction of ERK phosphorylation induced by MCAO in the prefrontal cortex (*p* value < 0.05, 0.01, and 0.001, respectively; compared to the HCHF+MCAO+vehicle group). In addition, donepezil and all doses of PMG also significantly improved the decrease in the expression of ERK phosphorylation induced by MCAO in the hippocampus of MetS rats (*p* value < 0.01, 0.001, 0.001, and 0.001, respectively; compared to the HCHF+MCAO+vehicle group).

### 3.5. The Alterations of IL-6 Expression in Prefrontal Cortex and Hippocampus

**Figure 10:**
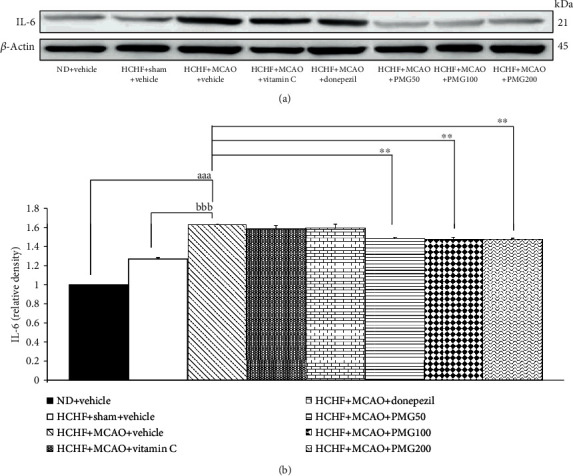
Effect of PMG on the expression of IL-6 in the prefrontal cortex was detected by Western blotting. (a) Representative Western blot showing the levels of total IL-6. (b) Relative density of total IL-6. Data are presented as mean ± SEM (*n* = 6/group). ^aaa^*p* value < 0.001; compared between naïve control which received a normal diet and vehicle and MetS+MCAO rats which received HCHF, MCAO, and vehicle. ^bbb^*p* value < 0.001; compared between sham rats which received HCHF, sham operation, and vehicle and MetS+MCAO rats which received HCHF, MCAO, and vehicle. ^∗∗^*p* value < 0.01; compared to MetS+MCAO rats which received HCHF, MCAO, and vehicle. ND: normal diet; HCHF: high-carbohydrate high-fat diet; MetS: metabolic syndrome; MCAO: middle cerebral artery occlusion; Vitamin C: vitamin C at a dose of 250 mg·kg^−1^ BW; Donepezil: donepezil at a dose of 3 mg·kg^−1^ BW; PMG50, PMG100, and PMG200: the phytosomes containing the combined extract of mulberry fruit and ginger at doses of 50, 100, and 200 mg·kg^−1^ BW, respectively.

**Figure 11:**
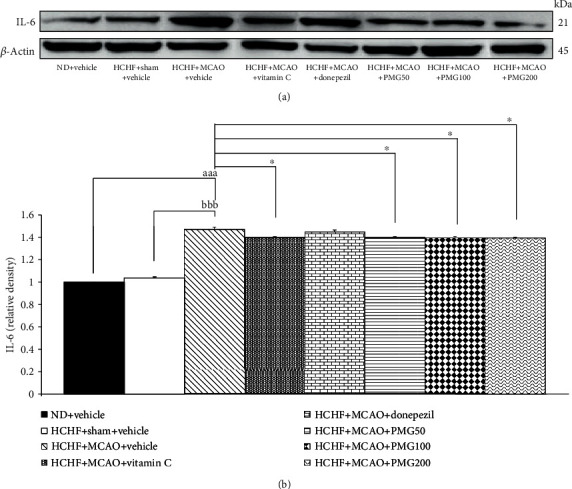
Effect of PMG on the expression of IL-6 in the hippocampus was detected by Western blotting. (a) Representative Western blot showing the levels of total IL-6. (b) Relative density of total IL-6. Data are presented as mean ± SEM (*n* = 6/group). ^aaa^*p* value < 0.001; compared between naïve control which received a normal diet and vehicle and MetS+MCAO rats which received HCHF, MCAO, and vehicle. ^bbb^*p* value < 0.001; compared between sham rats which received HCHF, sham operation, and vehicle and MetS+MCAO rats which received HCHF, MCAO, and vehicle. ^∗^*p* value < 0.05; compared to MetS+MCAO rats which received HCHF, MCAO, and vehicle. ND: normal diet; HCHF: high-carbohydrate high-fat diet; MetS: metabolic syndrome; MCAO: middle cerebral artery occlusion; Vitamin C: vitamin C at a dose of 250 mg·kg^−1^ BW; Donepezil: donepezil at a dose of 3 mg·kg^−1^ BW; PMG50, PMG100, and PMG200: the phytosomes containing the combined extract of mulberry fruit and ginger at doses of 50, 100, and 200 mg·kg^−1^ BW, respectively.

Since neuroinflammation plays a crucial role on cognitive impairment [31], we also investigated the effect of PMG on proinflammatory cytokines such as IL-6 in the prefrontal cortex and the hippocampus, and the results are shown in Figures 10 and 11. The results revealed that MCAO significantly increased the expression of IL-6 in both the prefrontal cortex and the hippocampus (all *p* value < 0.001; compared to the HCHF+sham operation+vehicle group) of MetS rats. Vitamin C significantly suppressed IL-6 expression only in the hippocampus (*p* value < 0.05; compared to the HCHF+MCAO+vehicle group). Interestingly, PMG treatments significantly suppressed the expressions of IL-6 in the prefrontal cortex (all *p* value < 0.01; compared to the HCHF+MCAO+vehicle group) and the hippocampus (all *p* value < 0.05; compared to the HCHF+MCAO+vehicle group).

## 4. Discussion

The current results have demonstrated the cognitive enhancing effect of PMG together with the increase in neuron density in both the cerebral cortex and the hippocampus. In addition, the reduction of AChE activity, MDA level, and IL-6 expression and the increase in SOD, CAT, and GSH-Px activities and ERK phosphorylation in both the cerebral cortex and the hippocampus of MetS rats were observed.

The cognitive enhancing effect of PMG observed in this study corresponded with the previous study which showed the cognitive enhancing effect following cerebral ischemia [9, 11]. Currently, it has been demonstrated that memory function is associated with the changes in the cholinergic system, ERK signal transduction, and brain structural changes in the hippocampus [6, 7]. A recent study has revealed that not only the hippocampus but also the prefrontal cortex plays crucial roles in spatial memory [32]. Our data also supported this information. MCAO induced memory impairment by modifying the aforementioned parameters in both the cerebral cortex and the hippocampus of MetS rats. In addition, our data also demonstrated that the alteration of retention time which was obtained from the probe trial phase of the Morris water maze test is more sensitive to PMG than the change of escape latency which was obtained from the acquisition phase of the Morris water maze test. It has been proposed that the learning process and retrieval process involve different pathways. The Dentate Gyrus-Perforant Pathway (DG-PP) has been revealed to play a role on learning or encoding of information, whereas the CA3-Mossy Fiber Pathway (CA3-MF) has been reported to play a role on memory retrieval [33]. Therefore, our data suggest that the CA3-Mossy Fiber Pathway (CA3-MF) may be more sensitive to PMG than the Dentate Gyrus-Perforant Pathway (DG-PP); therefore, the positive modulation effect of PMG on the CA3-MF pathway may be higher than that on the DG-PP pathway. Therefore, the positive modulation effect on retention time has been observed sooner than the positive modulation effect on escape latency. However, this issue still required further investigation to confirm the suggestion. One of the limitations of this study was the inability to determine all changes of the observed parameters contributing to the crucial roles on brain plasticity, especially the signal transduction process and memory performance in all subregions of the hippocampus. In addition, no changes in synaptic density and mossy fiber pathway were determined.

It has been demonstrated that neurodegeneration is associated with memory impairment [34]. Proinflammatory cytokines such as IL-6 and oxidative stress play pivotal roles in neurodegeneration [7, 34, 35] and memory impairment. We have found that PMG induces the increase in neuron density in the cerebral cortex and the hippocampus together with the reduction of MDA and IL-6 but an increase in SOD, CAT and GSH-Px. Therefore, we suggest that PMG may increase the activities of all scavenger enzymes mentioned earlier resulting in the reduction of the MDA level. Both the reduction of oxidative stress manifesting by the decrease in MDA and inflammation manifesting by the reduction of IL-6 contribute to the important roles on the improvement of neurodegeneration resulting in the increase of neuron density and memory.

It has been reported that ACh can improve memory via the interaction with the muscarinic receptor which in turn stimulates signal transduction via the ERK pathway [36] and suppresses inflammation [37]. Our results clearly demonstrate that PMG suppresses AChE in both the cerebral cortex and the hippocampus. Therefore, we suggest that AChE suppression induced by PMG leads to the increase in ACh and the stimulation of the ERK pathway or the phosphorylation of ERK leads to the improvement of memory. In addition, the inhibition of AChE induced by PMG also suppresses inflammation giving rise to improved memory deficit.

Due to the cognitive enhancing effect together with the antioxidant and anti-inflammatory effects of polyphenols specially the anthocyanins and gingerol mentioned earlier [6, 8–11], it has been suggested that the cognitive enhancing effect of PMG may occur partly due to the effects of the absorbed polyphenols. The results obtained from this study failed to show the dose response study. A possible explanation might occur partly via the lack of a linear relationship between the concentrations of PMG and the observed parameters due to the multifactor involvement. It has been suggested that polyphenols are present in PMG and most of the absorbed polyphenols reach the large intestine and modify gut microbiota composition giving rise to the modulation of the gut-brain axis leading to the reduction of neurodegeneration and memory enhancement [38]. In addition, PMG contains numerous ingredients so the masking effect induced by increments of some ingredients during an increase in the administered dose might also blunt the effect of the active ingredients.

Taken all together, this study clearly reveals the cognitive enhancing effect of PMG. The possible underlying mechanism may occur partly via the improvement of cholinergic function which in turn increases the phosphorylation of the ERK signal molecule and via the improvement of oxidative stress status and inflammation leading to the improvement of brain plasticity and memory improvement.

## 5. Conclusion

**Figure 12:**
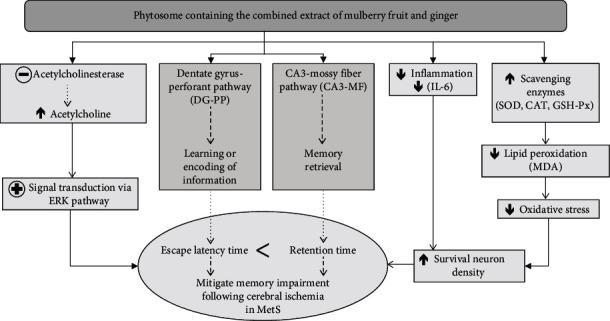
Schematic diagram demonstrating the neuroprotective effect of PMG against cognitive impairment following ischemic stroke in the animal model of ischemic stroke in MetS condition. ERK: extracellular signal-regulated kinase; IL-6: interleukin-6; MDA: malondialdehyde; SOD: superoxide dismutase; CAT: catalase; GSH-Px: glutathione peroxidase; MetS: metabolic syndrome.

This study is the first study to demonstrate the neuroprotective and cognitive enhancing effects of PMG. The possible underlying mechanisms might partly be through multitargets including the improvement of oxidative stress, inflammation, AChE activity, and ERK signal transduction as shown in Figure 12. Therefore, PMG can serve as functional ingredients for developing neuroprotectants and cognitive enhancers to treat cognitive impairment following ischemic stroke in MetS condition. However, subchronic toxicity studies are required in order to assure the safety of PMG consumption before moving forward to a clinical trial study.

## Figures and Tables

**Table 1:** The effect of PMG on acetylcholinesterase activity and oxidative stress alterations in the prefrontal cortex.

Treatment groups	AChE (nmol/min·mg protein)	MDA (ng/mg protein)	SOD (units/mg protein)	CAT (units/mg protein)	GSH-Px (units/mg protein)
ND+vehicle	0.15 ± 0.02	0.55 ± 0.12	17.14 ± 1.17	32.63 ± 4.20	8.93 ± 0.18
HCHF+sham+vehicle	0.18 ± 0.03	0.60 ± 0.04	16.66 ± 1.45	29.39 ± 3.73	8.50 ± 0.29
HCHF+MCAO+vehicle	0.39 ± 0.01^aaa,bbb^	3.31 ± 0.17^aaa,bbb^	6.53 ± 0.71^aa,bb^	22.46 ± 2.31^aa,bb^	3.97 ± 0.06^aaa,bbb^
HCHF+MCAO+vitamin C	0.31 ± 0.02	1.25 ± 0.36^∗∗∗^	11.39 ± 2.99^∗∗^	27.27 ± 1.80	5.31 ± 0.28^∗^
HCHF+MCAO+donepezil	0.21 ± 0.01^∗∗^	2.99 ± 0.10	8.42 ± 0.68	22.52 ± 4.95	4.06 ± 0.64
HCHF+MCAO+PMG50	0.25 ± 0.02^∗^	0.70 ± 0.05^∗∗∗^	14.96 ± 1.15^∗∗∗^	29.14 ± 2.34	6.37 ± 1.06^∗∗∗^
HCHF+MCAO+PMG100	0.22 ± 0.01^∗∗^	0.68 ± 0.08^∗∗∗^	14.21 ± 1.62^∗∗∗^	29.10 ± 7.52^∗^	6.05 ± 1.00^∗∗∗^
HCHF+MCAO+PMG200	0.24 ± 0.01^∗^	0.62 ± 0.08^∗∗∗^	15.49 ± 1.04^∗∗∗^	31.16 ± 6.19^∗^	6.10 ± 0.94^∗∗∗^

Data are presented as mean ± SEM (*n* = 6/group). ^aa^*p* value < 0.01 and ^aaa^*p* value < 0.001; compared between control rats which received a normal diet and vehicle and MetS+MCAO rats which received HCHF, MCAO, and vehicle. ^bb^*p* value < 0.01 and ^bbb^*p* value < 0.001; compared between sham rats which received HCHF, sham operation, and vehicle and MetS+MCAO rats which received HCHF, MCAO, and vehicle. ^∗^*p* value < 0.05, ^∗∗^*p* value < 0.01, and ^∗∗∗^*p* value < 0.001; compared to MetS+MCAO rats which received HCHF, MCAO, and vehicle. ND: normal diet; HCHF: high-carbohydrate high-fat diet; MetS: metabolic syndrome; MCAO: middle cerebral artery occlusion; Vitamin C: vitamin C at a dose of 250 mg·kg^−1^ BW; Donepezil: donepezil at a dose of 3 mg·kg^−1^ BW; PMG50, PMG100, and PMG200: the phytosomes containing the combined extract of mulberry fruit and ginger at doses of 50, 100, and 200 mg·kg^−1^ BW, respectively.

**Table 2:** The effect of PMG on acetylcholinesterase activity and oxidative stress markers in hippocampus.

Treatment groups	AChE (nmol/min·mg protein)	MDA (ng/mg protein)	SOD (units/mg protein)	CAT (units/mg protein)	GSH-Px (units/mg protein)
ND+vehicle	0.18 ± 0.01	0.50 ± 0.07	16.29 ± 0.84	69.63 ± 9.55	8.85 ± 0.54
HCHF+sham+vehicle	0.20 ± 0.01	0.55 ± 0.04	15.45 ± 0.24	66.49 ± 10.23	8.41 ± 0.38
HCHF+MCAO+vehicle	0.35 ± 0.01^aaa,bbb^	3.06 ± 0.28^aaa,bbb^	7.31 ± 0.46^aaa,bbb^	46.69 ± 1.10^aaa,bbb^	3.96 ± 0.20^aaa,bbb^
HCHF+MCAO+vitamin C	0.31 ± 0.01	1.08 ± 0.12^∗∗∗^	9.28 ± 1.12^∗∗^	51.86 ± 6.13^∗^	4.77 ± 0.93^∗^
HCHF+MCAO+donepezil	0.20 ± 0.03^∗∗∗^	2.82 ± 0.09	7.36 ± 1.01	47.46 ± 2.77	4.27 ± 0.27
HCHF+MCAO+PMG50	0.25 ± 0.01^∗∗^	0.63 ± 0.07^∗∗∗^	12.41 ± 0.95^∗∗∗^	48.54 ± 13.95	6.42 ± 0.57^∗∗∗^
HCHF+MCAO+PMG100	0.23 ± 0.01^∗∗∗^	0.63 ± 0.08^∗∗∗^	11.32 ± 1.50^∗∗∗^	60.52 ± 9.76^∗∗^	7.29 ± 1.13^∗∗∗^
HCHF+MCAO+PMG200	0.20 ± 0.01^∗∗∗^	0.54 ± 0.04^∗∗∗^	14.17 ± 1.64^∗∗∗^	62.86 ± 5.76^∗∗^	6.76 ± 0.46^∗∗∗^

Data are presented as mean ± SEM (*n* = 6/group). ^aaa^*p* value < 0.001; compared between control rats which received a normal diet and vehicle and MetS+MCAO rats which received HCHF, MCAO, and vehicle. ^bbb^*p* value < 0.001; compared between sham rats which received HCHF, sham operation, and vehicle and MetS+MCAO rats which received HCHF, MCAO, and vehicle. ^∗^*p* value < 0.05, ^∗∗^*p* value < 0.01, and ^∗∗∗^*p* value < 0.001; compared to MetS+MCAO rats which received HCHF, MCAO, and vehicle. ND: normal diet; HCHF: high-carbohydrate high-fat diet; MetS: metabolic syndrome; MCAO: middle cerebral artery occlusion; Vitamin C: vitamin C at a dose of 250 mg·kg^−1^ BW; Donepezil: donepezil at a dose of 3 mg·kg^−1^ BW; PMG50, PMG100, and PMG200: the phytosomes containing the combined extract of mulberry fruit and ginger at doses of 50, 100, and 200 mg·kg^−1^ BW, respectively.

## Data Availability

The data are available and will be provided on request because during this period, all data are in the process of petty patent registration.
